# Reduction in oral corticosteroid use in patients receiving omalizumab for allergic asthma in the real-world setting

**DOI:** 10.1186/1710-1492-9-47

**Published:** 2013-12-04

**Authors:** Gert-Jan Braunstahl, Jan Chlumský, Guy Peachey, Chien-Wei Chen

**Affiliations:** 1 Department of Pulmonary Medicine, Sint Franciscus Gasthuis, Kleiweg 500, 3045 PM, Rotterdam, The Netherlands; 2 Department of Pulmonary Disease, Thomayer Hospital, Charles University, Prague, Czech Republic; 3 Novartis Pharmaceuticals UK Limited, Horsham, West Sussex, UK; 4 Novartis Pharmaceuticals Corporation, East Hanover, NJ, USA

**Keywords:** Anti-immunoglobulin E, Oral corticosteroid use, Omalizumab, Registry, Uncontrolled persistent allergic asthma

## Abstract

**Background:**

Oral corticosteroids (OCS) are commonly administered in patients with severe persistent allergic asthma. Despite their efficacy, they are associated with a wide variety of adverse events. The eXpeRience registry was set up to investigate real-world outcomes among patients receiving omalizumab for the treatment of uncontrolled allergic asthma. Here, we present the effect of omalizumab treatment on OCS use.

**Methods:**

eXpeRience was a 2-year, multinational, non-interventional, observational registry of patients receiving omalizumab for uncontrolled allergic asthma. OCS use (proportion of patients on maintenance OCS, mean total daily OCS dose and change in status of OCS therapy) was assessed at baseline, 16 weeks, and 8, 12, 18, and 24 months after the initiation of omalizumab. Response to omalizumab was assessed using the physician’s Global Evaluation of Treatment Effectiveness (GETE) at approximately Week 16. Safety data were also recorded.

**Results:**

A total of 943 patients (mean age, 45 years; female, 64.9%) were enrolled in the registry, 263 of whom were receiving maintenance OCS at baseline. The proportion of patients taking maintenance OCS was markedly lower at Months 12 (16.1%) and 24 (14.2%) than at baseline (28.6%; intent-to-treat population). GETE status was determined in 915 patients receiving omalizumab: 64.2% were responders (excellent or good response), 30.7% were non-responders (moderate, poor or worsening response); 5.1% had no assessment. The frequency of serious adverse events was comparable to that seen in controlled trials of omalizumab.

**Conclusions:**

Omalizumab use is associated with an OCS-sparing effect in patients with uncontrolled persistent allergic asthma in the real-world setting.

## Background

Patients with allergic asthma are often inadequately controlled despite treatment with high-dose inhaled corticosteroids (ICS) and long-acting β_2_-agonists (LABA) [1,2]. Oral corticosteroids (OCS) are commonly administered to suppress airway inflammation and improve asthma control in these patients; however, their long-term use is associated with significant adverse effects, such as diabetes, osteoporosis and cataract formation, placing a major burden on patients and healthcare resources [3-6]. Interventions that allow OCS treatment to be reduced or withdrawn completely are likely to benefit patients receiving these agents for the treatment of asthma.

Omalizumab, a humanized anti-immunoglobulin E (IgE) monoclonal antibody, is approved for the treatment of patients with uncontrolled moderate-to-severe (US) or severe (EU) persistent allergic (IgE-mediated) asthma [7,8]. Omalizumab has been shown to reduce asthma exacerbations and hospital visits, as well as corticosteroid use, in patients with allergic asthma [9-11]. Omalizumab has also been shown to have a direct OCS-sparing effect in a 32-week randomized, open-label study in adolescents and adults (12–75 years) with severe asthma [12], as well as in a 16-week uncontrolled therapeutic trial in children (median age 12 years) [13].

eXpeRience was an international registry initiated to evaluate outcomes in patients receiving omalizumab for uncontrolled persistent allergic asthma in ‘real-world’ clinical practice. The primary results, published previously [14,15], showed that omalizumab was associated with improvements in clinical outcomes such as asthma exacerbations and objective measures of asthma control. Here, we evaluate the real-world effect of omalizumab treatment on the use of OCS over a 2-year period.

## Methods

eXpeRience was a multinational, non-interventional, observational registry established to collect data on the real-world effectiveness and safety of omalizumab therapy during routine clinical practice in patients with uncontrolled persistent allergic (IgE-mediated) asthma. The registry design has been published previously [15].

Briefly, the registry included male and female patients with uncontrolled persistent allergic asthma who had commenced omalizumab treatment within the previous 15 weeks. Patients from 14 countries in Europe, America and Asia were enrolled, and were followed for up to 2 years after initiation of omalizumab. After entry into the registry, data were collected prospectively at approximately 16 weeks and at 8, 12, 18 and 24 months after initiation of omalizumab treatment, with a minimum requirement of two data collections per year.

Treatment and follow-up of patients was at the discretion of the treating physician, according to local medical practice and label/reimbursement guidelines. The registry design and amendments were reviewed by independent ethics committees or institutional review boards at each participating centre, as required.

### Registry assessments

Data on OCS use were collected at each pre-determined time-point. The variables evaluated included: proportion of patients receiving OCS as maintenance therapy; total daily OCS dose; change from baseline in OCS dose; number of patients in whom OCS therapy was stopped, reduced (without stopping), or increased as compared with baseline; time to reduction in OCS dose or stopping OCS therapy. Data on ICS use were also collected at each time-point, including: total daily ICS dose; change from baseline in ICS dose; number of patients in whom ICS therapy was stopped, reduced (without stopping), or increased as compared with baseline.

Response to omalizumab was assessed using the physician’s Global Evaluation of Treatment Effectiveness (GETE) at approximately Week 16 after the initiation of treatment. OCS use among omalizumab responders (i.e. those with an “excellent” or “good” response by GETE) and non-responders (i.e. “moderate” or “poor” response, or “worsening” asthma) was evaluated. OCS doses were converted to prednisolone equivalents (1 mg prednisone = 1 mg prednisolone; 1 mg methylprednisolone = 1.25 mg prednisolone).

Safety was assessed by recording the nature and frequency of serious adverse events (SAEs) that occurred during the registry, which were followed until resolution.

### Statistical analysis

All efficacy analyses reported are based on the intent-to-treat (ITT) population, consisting of all randomized patients who had at least one post-baseline efficacy assessment. All safety analyses are based on the safety population, which included all patients who received at least one dose of omalizumab and had at least one post-baseline safety assessment.

Statistical analyses were mainly descriptive. Summary statistics describing change from baseline in OCS dose, reduction or cessation of OCS treatment, and time to reduction were calculated for all patients receiving maintenance OCS therapy at baseline, and for GETE-defined responders and non-responders.

## Results

### Patient disposition and baseline characteristics

A total of 943 patients were included in the eXpeRience registry. Of these patients, 694 (73.6%) completed the registry and 157 (16.6%) discontinued; status was unknown for 92 (9.8%). The most common reasons for discontinuation were loss to follow-up (n = 52; 5.5%) and withdrawal of consent (n = 27; 2.9%) [14]. Demographic and clinical characteristics of the patients receiving OCS at baseline were comparable with the overall population (Table 1; [14]). The ITT population included 916 patients and the safety population included 925 patients. Of the 263 patients receiving OCS at baseline, the ITT population included 246 patients and the safety population included 263 patients.

**Table 1:** Baseline demographics and clinical characteristics of patients in the eXpeRience registry (safety population)

**Variable**	**Patients on OCS at baseline** **(n = 263)**	**Overall population** **(n = 925)**
**Mean age,** years (SD)	46.0 (13.3)	45.0 (15.0)
**Female,** n (%)	169 (64.3)	600 (64.9)
**Race,** n (%)		
Caucasian	246 (93.5)	855 (92.4)
Others	17 (6.5)	70 (7.6)
**Mean duration of allergic asthma**, years **(SD)**	20.3 (13.6) [n = 261]	19.4 (13.6)
**Positive skin-prick test/RAST for perennial aeroallergens,** n (%)	232 (88.2)	816 (88.2)^†^
**History of seasonal allergy,** n (%)	177 (67.3)	587 (63.5)^†^
**Smoking history,** n (%)		
Never smoked	202 (76.8)	719 (77.7)^‡^
Ex-smoker	52 (19.8)	173 (18.7)
Current smoker	9 (3.4)	30 (3.2)
**Asthma clinical symptoms, n (%)**		
Daytime asthma symptoms	245 (93.2)	838 (90.6)
Limitations of activities	239 (90.9)	795 (85.9)
Nocturnal symptoms/awakenings	218 (82.9)	737 (79.7)
**Asthma control (investigator assessment), n (%)**		
Controlled	3 (1.1)	13 (1.4)^†^
Partly controlled	42 (16.0)	215 (23.2)
Uncontrolled	218 (82.9)	693 (74.9)

^†^Data missing for one patient; ^‡^Data missing for three patients.

RAST, radioallergosorbent test; SD, standard deviation.

### OCS use

**Figure 1:**
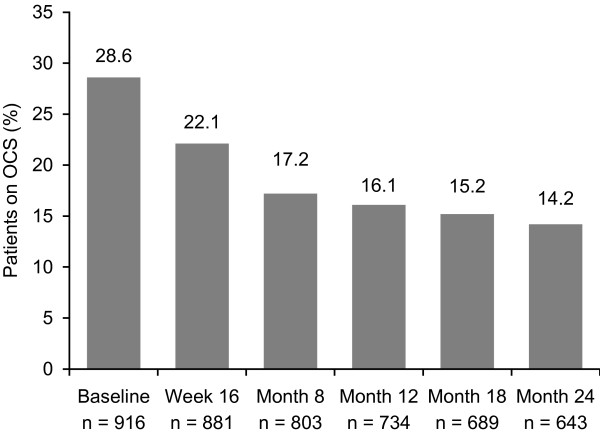
**Proportion of patients on maintenance OCS.** n = Number of evaluable patients at each time point. OCS, oral corticosteroids.

**Figure 2:**
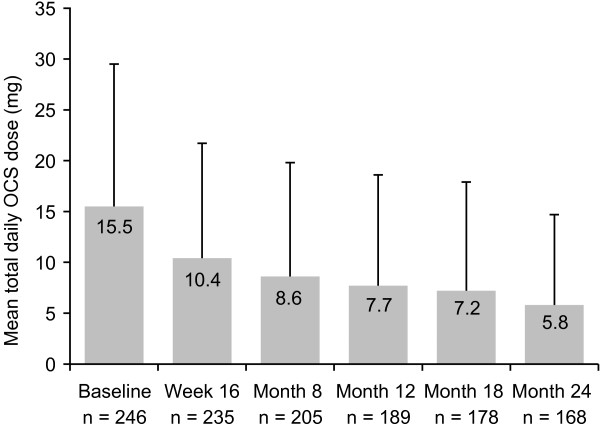
**Mean total daily OCS dose (prednisolone equivalent) (ITT population).** n = number of evaluable patients at each time-point. OCS, oral corticosteroids; SD, standard deviation; ITT, intent-to-treat. Error bars represent SD. Post-baseline data include doses of zero for patients no longer receiving OCS.

The most commonly used OCS was prednisone (n = 131 at baseline; 49.8% of all patients receiving OCS). The proportion of patients receiving OCS was lower at Months 12 (16.1%) and 24 (14.2%) than at baseline (28.6%) (Figure 1). The mean total daily OCS dose (prednisolone equivalent) decreased between baseline (15.5 mg) and Month 12 (7.7 mg), and continued to decrease between Months 12 and 24 (5.8 mg) (Figure 2).

**Figure 3:**
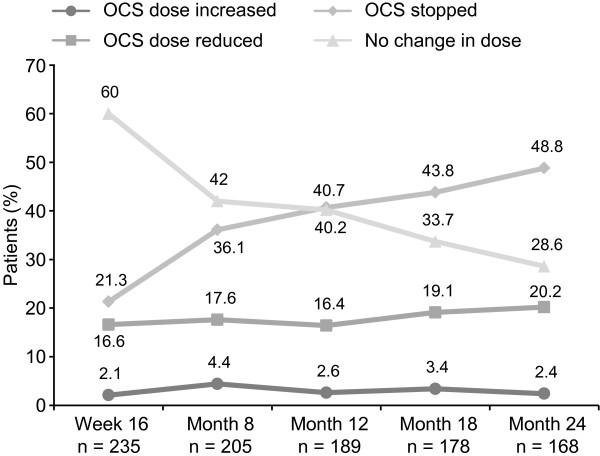
**Change in status of OCS therapy in patients receiving maintenance OCS at baseline (n = 246; ITT population).** n = Number of evaluable patients at each time-point. OCS, oral corticosteroids; ITT, intent-to-treat. Post-baseline data include doses of zero for patients no longer receiving OCS. Data at each time-point are relative to baseline.

Among patients receiving OCS at baseline, there was a reduction in or discontinuation of OCS treatment in 57.1% and 69.0% of patients at Months 12 and 24, respectively (Figure 3). The mean (SD) time to reduction or discontinuation of OCS was 198.5 (114.29) days and 291.2 (210.86) days, assessed at Months 12 and 24, respectively. Five patients (2.6%) at Month 12 and four patients (2.4%) at Month 24 had an increase in OCS dose.

### OCS use according to response

Of the 915 patients assessed using the GETE, 64.2% were responders (excellent, 11.4%; good, 52.8%) and 30.7% were non-responders (moderate, 23.4%; poor, 6.8%; worsening of asthma, 0.5%) based on GETE status; 5.1% had no assessment. Following the GETE assessment, the vast majority of responders (98.1%) and ‘moderate’ non-responders (96.3%) continued with treatment.

Among responders, the proportion of patients on maintenance OCS treatment decreased from 28.1% at baseline to 13.8% at Month 12 and 12.1% at Month 24. Mean (SD) total daily dose (prednisolone equivalent) decreased from 15.5 (14.63) mg at baseline to 6.5 (10.13) mg at Month 12, and 5.1 (8.94) mg at Month 24.

Among GETE non-responders, the proportion of patients on maintenance OCS treatment decreased from 28.5% at baseline to 20.9% at Month 12 and 17.9% at Month 24. Mean (SD) total daily dose (prednisolone equivalent) decreased from 14.7 (10.92) mg at baseline to 11.3 (12.85) mg at Month 12, and 8.1 (8.99) mg at Month 24.

### ICS use

At baseline, 895 of 916 patients (98%) were receiving ICS maintenance therapy. Among patients who provided ICS information, 173 of 705 (24.5%) and 182 of 613 (29.7%) had stopped or reduced ICS use at Months 12 and 24, respectively. The majority of patients did not change their ICS dose (495 [70.2%] at Month 12 and 389 [63.5%] at Month 24), while 37 patients (5.2%) and 42 patients (6.9%) had an increase in dose at Months 12 and 24, respectively. Mean (SD) total daily ICS doses (beclomethasone equivalent) decreased from 1675 (947) μg at baseline to 1461 (950) at Month 12 and 1381 (961) at Month 24. Mean (SD) percentage reductions in ICS dose from baseline were 9.6% (49.8) at Month 12 and 11.8% (57.1) at Month 24.

### ICS use according to response

Among GETE responders, the mean (SD) ICS dose decreased from baseline by 266 (759) μg at Month 12 and 313 (819) μg at Month 24, with mean (SD) percentage reductions of 12.9 (41.8) and 14.8 (47.4), respectively. Among the responders, 133 of 487 (27.3%) and 137 of 435 (31.5%) had stopped or reduced ICS use at Months 12 and 24, respectively.

Among GETE non-responders, the mean (SD) ICS dose decreased from baseline by 134 (847) μg at Month 12 and 246 (1122) μg at Month 24, with mean (SD) percentage reductions of 2.8 (65.9) and 4.8 (77.9), respectively. Among the non-responders, 40 of 200 (20.0%) and 44 of 164 (26.8%) had stopped or reduced ICS use at Months 12 and 24, respectively.

### Safety

Detailed safety findings from the registry have been published previously [14]. Briefly, a total of 64 patients (6.9%) reported 150 SAEs. Of these, 25 SAEs (16.7%) were suspected to be related to omalizumab. The most common SAE was asthma (n = 32, 3.5%), followed by dyspnoea and pneumonia (both n = 7, 0.8%). Nine deaths occurred during the registry; none were suspected to be related to omalizumab (causes of death have been described previously [14]).

## Discussion

In this registry, omalizumab was associated with a reduction in maintenance OCS use in patients with uncontrolled persistent allergic asthma over a 2-year treatment period in a real-world setting. Approximately half of the patients on maintenance OCS at baseline were able to stop or reduce their OCS dose. In addition, there were also reductions in maintenance ICS use over the 2 years of the study. We believe that the observed reductions in OCS and ICS use reflect improved asthma control during treatment with omalizumab, and are likely to be associated with a reduction in the risk of steroid-related adverse effects. Reductions in OCS use and mean total daily OCS dose were greater in patients classified as responders to omalizumab treatment than in non-responders. Nevertheless, a reduction in OCS use and mean total daily OCS dose was seen in non-responders, possibly due to the fact that the definition of non-responders included patients with a moderate response to omalizumab (as well as those with a poor or worsening response). This is highlighted by the vast majority of moderate ‘non-responders’ continuing with treatment, following the GETE assessment.

Corticosteroids are widely prescribed to treat inflammatory conditions, including asthma, for which they are often the mainstay of treatment [5]. Patients with uncontrolled severe asthma may require long-term maintenance therapy with OCS. However, such use is associated with serious long-term adverse effects such as hypothalamic-pituitary-adrenal axis suppression, impaired glucose tolerance and diabetes, osteoporosis, hypertension, and cataract formation. There is therefore a need for therapies that improve outcomes, have acceptable safety and tolerability profiles, while allowing reductions in OCS use [12].

Despite the observed reductions in OCS use, patients enrolled in the eXpeRience registry had fewer clinically significant asthma exacerbations after 12 or 24 months’ treatment with omalizumab (annualized mean 1.0 and 0.6, respectively) compared with pre-treatment values (annualized mean 4.9) [14]. Relatedly, omalizumab was also associated with reductions in healthcare utilization (hospitalizations, emergency room visits and unscheduled doctor visits), from 6.2 during the 12-month pre-treatment period to 1.0 per year at Month 12 and 0.5 per year at Month 24 [14]. Annualized numbers of days of absence from work and school due to asthma were also lower at Month 12 (3.5 and 1.6 days, respectively) and Month 24 (1.0 and 1.9 days, respectively) than during the 12-month pre-treatment period (26.4 and 20.7 days, respectively) [14].

Our findings are in agreement with those of other real-life studies of omalizumab. A pooled analysis of data from French and German patients (n = 346) with severe persistent allergic asthma showed that omalizumab treatment for at least 16 weeks was associated with a reduction or discontinuation of OCS in 50% of patients receiving OCS at baseline (n = 84/166) [16]. The mean reduction in daily OCS dose from baseline was 74.3% [16]. An additional French historic-prospective study showed that 48.1% of patients reduced or discontinued maintenance OCS over a period of ≥5 months of omalizumab treatment [17].

Decreased use of OCS subsequent to treatment with omalizumab has also been shown in observational studies conducted in Italy [18], Belgium [19], Israel [20] and the United Kingdom [21]. These studies enrolled between 22 and 142 patients, who were followed up for between 16 and 52 weeks. Between 20% and 70% of patients taking OCS at baseline were able to stop or reduce treatment, and showed meaningful reductions in exacerbations rates.

The results of this analysis indicate that a significant proportion of patients on omalizumab therapy were classed as responders by physician’s GETE at Week 16. These results are consistent with omalizumab clinical trials. In an early randomized controlled study, 53% of omalizumab-treated patients had an excellent/good response to omalizumab, compared with 33% for placebo [22]; in a more recent study, 72.8% in the omalizumab plus optimized asthma therapy (OAT) group responded, compared to 31.2% for OAT alone [23]. However, not all responses to omalizumab are achieved within the first 16 weeks of therapy, with some patients taking longer to respond [23].

Bousquet et al. demonstrated that the physician’s GETE at Week 16 is an effective predictor of longer-term outcomes, including exacerbation rates, overall asthma control and unscheduled medical interventions [24]. Subsequently, Bousquet et al. also showed that the majority of patients classified as responders or non-responders at Week 16 have the same classification at Week 32 [23]. Consistent with the findings of Bousquet et al. [23,24], the present study also indicated that asthma control (as reflected in OCS use) is improved to a greater extent among responders to omalizumab, compared with non-responders, and that these improvements (and the differences between responders and non-responders) persist between Week 16 and 2 years.

Most of the observational studies conducted with omalizumab support a reduction in maintenance OCS dose to improve asthma management. However, in rare cases, patients receiving omalizumab may present with systemic hypereosinophilic syndrome or allergic eosinophilic granulomatous vasculitis (Churg-Strauss syndrome) [25], and these events are usually, but not always, associated with a reduction in OCS dose. We did not observe any cases of either hypereosinophilic syndrome or Churg-Strauss syndrome in the eXpeRience registry, but we believe that clinicians attempting OCS reduction or withdrawal in patients with allergic asthma should be aware of this.

Despite our positive findings, it is important to recognize the limitations of observational studies, namely the lack of a control group and the open-label design.

## Conclusions

In conclusion, this 2-year, international and observational registry, conducted in a real-life setting, confirms that omalizumab is associated with OCS-sparing effects in patients with uncontrolled persistent allergic (IgE-mediated) asthma.

## Abbreviations

OCS: Oral corticosteroids; GETE: Global Evaluation of Treatment Effectiveness; ICS: Inhaled corticosteroids; LABA: Long-acting β_2_-agonists; IgE: Immunoglobulin E; SAEs: Serious adverse events; ITT: Intent-to-treat; OAT: Optimized asthma therapy.

## Competing interests

G-JB has received grant/research support for consultations and/or speaking at conferences from Novartis, GSK, AstraZeneca, and MSD.

JC has received lecture fees from Novartis, and has been an investigator in studies sponsored by several other pharmaceutical companies. C-WC and GP are Novartis employees.

## Authors’ contributions

G-JB has contributed to the enrolment of patients, data capture and processing and has reviewed and commented various drafts of the manuscript. JC has contributed to enrolment of patients and has reviewed and commented various drafts of the manuscript. GP is a clinical lead accountable for the conduct and reporting of the registry; oversight of all operational aspects of the registry (e.g. patient enrolment; document management, etc.); scientific review of registry data, and lead author of the clinical study reports (interim and final). C-WC has contributed to data analysis. All authors read and approved the final manuscript.
